# The Unlikely Suspect: A Case Report of New-Onset Hyperthyroidism Due to Graves' Disease in an 89-Year-Old Gentleman and Review of Literature

**DOI:** 10.7759/cureus.21546

**Published:** 2022-01-24

**Authors:** Pranjali Sharma

**Affiliations:** 1 Endocrinology, Parkview Medical Center, Pueblo, USA

**Keywords:** hyperthyroidism management, thyroid pathology, thyroid stimulating immunoglobulin tsi, elderly population, hyperthyroidism, graves´disease

## Abstract

Thyroid dysfunction in the elderly commonly manifests as hypothyroidism. With advancing age, toxic nodules are the more common cause of hyperthyroidism as compared to Graves' disease. Due to the lack of classical symptoms of hyperthyroidism in the elderly, the diagnosis can often be delayed. Previously, an 82-year-old gentleman with oropharyngeal dysphagia due to Graves' disease was the oldest reported case with atypical symptoms. We report a case of an 89-year-old gentleman with no prior history of thyroid disease, who presented with non-specific gastrointestinal symptoms that ultimately led to the diagnosis of hyperthyroidism secondary to Graves' disease. We also review the available literature regarding the pathophysiology, clinical presentation, and management of hyperthyroidism and Graves' disease in the elderly.

## Introduction

Thyroid dysfunction in the elderly is commonly manifested as hypothyroidism. The prevalence of hyperthyroidism in the elderly is approximately 0.5-3%, of which 10-15% of hyperthyroid patients are above 60 years of age [1]. Graves' disease is the most common cause of hyperthyroidism, accounting for 60-80% of all cases of hyperthyroidism and primarily occurs between 20 and 50 years of age. It is more common in women than men [2]. With advancing age, the incidence of toxic multinodular goiter increases and Graves' disease becomes increasingly uncommon [1]. The exact prevalence of Graves' disease in the elderly is not known but is primarily driven by the previous history of the same [3]. The oldest reported case of new-onset Graves' hyperthyroidism was an 82-year-old gentleman with oropharyngeal dysphagia [4]. We report a case of an 89-year-old gentleman with gastrointestinal symptoms leading to the diagnosis of new-onset hyperthyroidism secondary to Graves' disease.

## Case presentation

An 89-year-old gentleman with a previous medical history of atrial fibrillation presented to his primary care physician with one month of nausea, vomiting, diarrhea, decreased appetite, and a 10 lb weight loss over several weeks. Additionally, his wife noted a change in mental status and reported increasing forgetfulness, tangential thoughts during the conversation, and increased sleep during the day. The patient also reported having vivid horrific dreams. He had no known personal or family history of thyroid disease. His medication list included metoprolol, lisinopril, amlodipine, furosemide, simvastatin, and apixaban. On physical examination, he had mild tachycardia (heart rate in the 100-110/min range), occasionally irregular rhythm, and bilateral coarse hand tremors. He did not have a goiter or abnormalities on eye examination. Initial stool cultures were negative while complete blood count was within normal limits. Metabolic panel showed glomerular filtration rate of 35 mL/min/1.73 m^2^ (normal range: >59 ml/min/1.73 m^2^) and creatinine level of 1.92 mg/dl (normal range: 0.76-1.27 mg/dl), suggesting acute kidney injury, not previously noted. The patient's symptoms were attributed to a gastrointestinal infection and neurological changes were attributed to the acute kidney injury. Therefore, he was advised to increase hydration.

His symptoms worsened over the next few weeks. A thyroid-stimulating hormone (TSH) level, sent as a part of the routine labs but not reviewed at the time, was abnormal (0.006 uIU/ml; normal range: 0.45-4.5 uIU/ml) (Table 1). A TSH level from a year prior was within the normal range. Repeat testing showed TSH level of 0.009 uIU/ml, free T4 level of 1.57 ng/dl (normal range: 0.82-1.77 ng/dl), total T4 level of 8.2 ug/dl (normal range: 4.5 - 12.0 ug/dl), and free T3 level of 4 pg/ml (normal range: 2.0-4.4 pg/ml), confirming subclinical hyperthyroidism (Table 1). Additional testing confirmed the etiology of Graves' disease due to elevated thyrotropin receptor antibody (TRAb) (6.21 IU/l; normal range: 0.00-1.75 IU/l) and thyroid-stimulating immunoglobulin (TSIg) (5.32 IU/l; normal range: 0.00-0.55 IU/l) (Table 2). Meanwhile, the patient reported worsening insomnia, additional weight loss, worsening atrial fibrillation and tremors, hot flashes, and skin dryness. However, updated TSH was 0.035 uIU/ml and the normal free T4 level was 1.23 ng/dl (Table 1). After discussion regarding therapy options, he opted to begin methimazole 5 mg daily. Thyroid function testing repeated six weeks later showed a TSH level of 1.91 uIU/ml with a normal free T4 level of 0.83 ng/dl (Table 1) without much change in symptomatology. At the three-month follow-up, TSH was 8.77 uIU/ml with a free T4 level of 0.82 ng/dl (Table 1) and the patient reported improved sleep, resolution of vivid dreams, improved palpitations and tremors, and no more hot flashes. Methimazole was decreased to 2.5 mg daily. The patient continues to follow up with endocrinology on a regular basis.

**Table 1:** Thyroid function tests This table includes all the thyroid tests done from the initial presentation to the current condition. Changes in methimazole dosing are also added. TSH - thyroid-stimulating hormone.

	TSH (uIU/ml)	Free T4 (ng/dl)	Free T3 (pg/ml)	Methimazole dose (mg)
Normal range	0.45-4.5	0.82-1.77	2.0-4.4	
Day 0	0.006			
Week 5	0.009	1.57	4	
Week 9	0.035	1.23		5 mg daily
Week 15	1.91	0.83		5 mg daily
Week 21	8.77	0.82		2.5 mg daily

**Table 2:** Antibody testing The elevated antibody titers led to the diagnosis of Graves' disease. TRAb - thyrotropin receptor antibody; TSIg - thyroid-stimulating immunoglobulin.

Antibody testing	Patient's results	Normal range
TRAb (IU/l)	6.21	0.00-1.75
TSIg (IU/l)	5.32	0.00-0.55

## Discussion

Graves' disease is an autoimmune disorder of the thyroid caused by TRAbs. These antibodies are produced by the lymphocytes in Graves' thyroid tissue [5]. TRAbs can be stimulating, blocking, or neutral antibodies. The stimulating TRAbs (also referred to as TSIg) stimulate the synthesis and activity of the sodium-iodide symporter and G proteins. This leads to increased iodine uptake, thyroid hormone synthesis and secretion, as well as increased thyroid cell proliferation and survival causing symptoms of hyperthyroidism [6,7]. Blocking TRAbs leads to hypothyroidism by binding to the TSH receptor and blocking the effect of TSH. Neutral antibodies bind to the hinge region of the TSH receptor and do not influence TSH action. They have been associated with thyroid cell stress and apoptosis but their clinical significance is so far unclear [8]. Patients with Graves' disease can have a mixture of stimulating and blocking antibodies and their balance decides whether the patient will have hyperthyroidism or hypothyroidism [9]. Our patient had high titers of the TRAb and TSIg leading to hyperthyroidism.

There is a complex relationship between age and TRAb titers and effects. In their study of 371 patients with Graves' disease, Aizawa et al. noted that while TRAb titers decreased progressively with age, there was no significant age-related difference in the mean TRAb titers [10]. They suggest that a decrease in TRAb production may play a minor role, if any, in causing mild hyperthyroidism in the elderly. They also hypothesized that TRAb in the elderly may have more blocking than stimulating activity as compared to younger patients. A recent study of 384 patients by Bano et al. showed that the effect of TRAb levels on relapse risk decreases with age, thereby making TRAb titers a less reliable predictor of relapse risk in the elderly [11]. A TRAb level > 10u/l caused milder thyrotoxicosis in patients above 55 years of age than in younger patients. Their post hoc analysis suggested that age does not affect TRAb bioactivity, but the authors suggested that alteration of the set point of the hypothalamic-pituitary-thyroid axis caused decreased thyroid gland response to TRAb stimulation. Our patient's antibody levels were more than three times and nine times the upper limit of normal for TRAb and TSIg, respectively. However, biochemically, these elevated titers only led to subclinical hyperthyroidism, in agreement with the above theory of decreased gland response.

Age-related changes in thyroid functions are a result of lower iodine intake in the elderly from dietary salt restriction and decreased absorption due to comorbidities, decreased thyroidal iodide uptake, decreased T4 release from the thyroid, decreased T4 clearance from decreased 5' deiodinase activity [1]. T3 levels only decline after 90 years of age due to lower T4 levels and decreased 5' deiodinase activity [1]. Inactive metabolite reverse T3 (rT3) increases with age [1]. Thyroid binding globulin (TBG) levels decrease with age, making the total T4 and total T3 level measurements less accurate; therefore, measuring free thyroid hormones is more useful in the elderly [1]. All these changes lead to milder biochemical hyperthyroidism in the elderly. This is another explanation for our patient's subclinical hyperthyroidism (low TSH and normal free T4 and T3) despite significantly elevated antibody titers.

Classic signs of hyperthyroidism secondary to Graves' disease are tremors, palpitations, weight loss, diarrhea, and heat intolerance. These symptoms tend to be absent in the elderly, who instead, can present with "apathetic thyrotoxicosis" - lethargy, depression, weight loss, and decreased appetite [1]. Some of these symptoms can be mistaken for an underlying malignancy. Persistent constipation rather than diarrhea is noted in the elderly. Cardiovascular morbidity and mortality due to atrial fibrillation, angina, and heart failure are high in this population. However, about 40% of patients have a heart rate of less than 100/min due to underlying conducting system disease [12]. Urinary frequency and nocturia also occur but the mechanism is still unclear [12]. Mental status changes such as dementia, agitation, and confusion can be mistaken for age-related changes [13]. Older patients are also less likely to have a goiter as compared to the younger patients, due to progressive fibrosis and atrophy of the thyroid gland leading to smaller thyroid volume [1,14]. Aggravation of postmenopausal osteoporosis and increased fracture risk is seen in elderly women with hyperthyroidism [1]. Graves' orbitopathy, while less common, tends to be severe in the elderly [15]. A retrospective study of 210 patients by Lin et al. demonstrated higher thyroid eye disease severity scores in patients above 50 years of age and suggested close monitoring of older patients with Graves' disease [16]. Pretibial myxedema, seen in <1 % of patients with Graves' disease, is more common in older patients [17].

Due to low clinical suspicion for hyperthyroidism and non-specificity of symptoms, a clinical diagnosis of hyperthyroidism is often missed initially, especially if there is no prior history of thyroid disease. Other diagnoses such as infections, cardiac events, cerebrovascular events, malignancies, or gastrointestinal diseases are first considered. Our patient did not have a history of thyroid disease. He presented with GI symptoms of nausea, vomiting, diarrhea, anorexia, and weight loss only. Cognitive changes were attributed to an acute kidney injury. He was evaluated and treated for a GI infection and the possibility of thyroid disease was not considered until several weeks later when the above symptoms did not improve and the previously missed abnormal TSH was noted. By then, he also had forgetfulness, change in sleep patterns, and worsening chronic tremors, which raised suspicion for a thyroid abnormality. He also did not have a goiter, which is typical in the elderly with hyperthyroidism. Curiously though, he had several symptoms of overt hyperthyroidism despite a picture of biochemical subclinical hyperthyroidism, some of which improved after initiation of methimazole treatment. This suggests that, while less common in the elderly, classic symptoms of hyperthyroidism do occur if the patient has biochemical abnormalities for a long enough period. Partial improvement in symptoms at three months indicates that classic hyperthyroid symptoms, once present, resolve slower than in younger patients.

Management of Graves' disease in the elderly includes antithyroid drugs (ATDs), radioactive iodine (RAI) ablation, or total thyroidectomy [13]. ATDs like methimazole or propylthiouracil are preferred as first-line therapy for mild cases of Graves' disease without cardiovascular complications. They can be administered as long-term maintenance therapy particularly in those with limited life expectancy, those unable to comply with radiation precautions, long-term facility residents, patients with urinary incontinence, or mental impairment [13]. Agranulocytosis is more common in elderly patients, particularly with propylthiouracil. It is commonly seen within three months of starting treatment [1].

RAI ablation is preferred over surgery for more severe cases of Graves' disease or in those who cannot tolerate ATD therapy, due to the higher risk of morbidity and mortality associated with surgery in the elderly [18]. RAI can be administered orally in the outpatient setting and is associated with very few side effects, making it a convenient treatment option for the elderly population. Precipitation of thyroid crisis during RAI ablation is possible and can be prevented by pre-treatment with ATDs. They are discontinued three to five days before therapy to ensure treatment efficacy and are restarted three to five days after RAI for continued prevention of thyroid storm [1]. The standard dose used in hyperthyroidism is 10-15 mCi of I-131. It has been suggested that larger doses should be administered to elderly patients, especially those with cardiovascular complications, to ensure rapid euthyroidism. Long-term risks of secondary cancers due to RAI are less important in elderly patients with Graves' disease [18]. Aiming for hypothyroidism through RAI ablation has shown to decrease mortality due to vascular complications [18].

Surgery is indicated in case of compressive symptoms due to a large goiter and if the patient is a good surgical candidate [1]. If surgery is elected as the definitive therapy of choice, pre-operative preparation with ATD, beta-blockers, and iodine is recommended to achieve euthyroidism if possible. Pre-operative preparation decreases the risk of precipitating thyroid storms during or post-surgery [13]. As our patient only had subclinical hyperthyroidism and no goiter, we initiated ATD therapy with methimazole 5 mg daily, which the patient has responded to.

## Conclusions

While Graves' disease is the most common cause of hyperthyroidism in young persons, toxic nodules are the leading cause of hyperthyroidism in the older population. This makes our 89-year-old gentleman's new diagnosis of Graves' disease all the more rare. To our knowledge, this is the oldest gentleman with atypical symptoms leading to a new diagnosis of Graves' disease.

Hyperthyroidism in the elderly can be a tricky diagnosis due to variable clinical presentation and the lack of classic features of thyrotoxicosis. ATDs and RAI ablation are preferred therapy options over surgery due to their good efficacy and safety profiles.
